# The interplay between left ventricular diastolic and right ventricular dysfunction: challenges in the interpretation of critical care echocardiography studies

**DOI:** 10.1186/s43044-023-00333-w

**Published:** 2023-01-24

**Authors:** Luigi La Via, Federica Merola, Giovanni Schembari, Calogero Liotta, Filippo Sanfilippo

**Affiliations:** 1 Department of Anesthesia and Intensive Care, “Policlinico-San Marco” University Hospital, Catania, Italy; 2 School of Anesthesia and Intensive Care, University of Catania, Catania, Italy; 3 School of Anesthesia and Intensive Care, University “Magna Graecia”, Catanzaro, Italy; 4 Department of Surgery and Medical-Surgical Specialties, University of Catania, Catania, Italy

**Keywords:** Guidelines, Recommendations, Critical care echocardiography, Left ventricle, Right ventricle

## Abstract

**Background:**

Sepsis is a leading cause of death and it is characterized not only by profound vasoplegia but also by myocardial dysfunction. Critical care echocardiography is the preferred modality for the initial assessment of the cause of shock. Moreover, it can be extremely helpful in the identification of progressing myocardial dysfunction during the course of sepsis, also known as septic cardiomyopathy.

**Main body:**

One of the issues in the identification of septic cardiomyopathy is that it can be manifest with different clinical phenotypes, from overt biventricular dysfunction to isolated left ventricular (LV) systolic and/or diastolic dysfunction, from right ventricular (RV) systolic dysfunction to RV failure and dilatation. However, the commonly used echocardiography parameters for the assessment of LV and/or RV function are not always entirely reliable. Indeed, these are influenced by variable preload and afterload conditions imposed by critical illness such as fluid shifts, sedation level and mechanical ventilation with positive pressure.

**Conclusions:**

Strain echocardiography is a promising tool for the early identification of myocardial dysfunction in the context of sepsis. Studies reporting data on strain echocardiography should be particularly detailed in order to increase the reproducibility of results and to favor comparison with future studies.

## Background

**Dear Editor,**

We read with interest the prospective study conducted by Bendary et al. [1] providing valuable hints on sepsis-induced cardiomyopathy [2, 3] and its impact on 30-day mortality, confirming the increased and valuable use of critical care echocardiography (CCE) [4]. In their study, the authors focused on the evaluation of right ventricular (RV) dysfunction, but conducted also a very detailed assessment of left ventricular (LV) systolic function and provided some hints on LV diastolic function reporting the E/e’ ratio values. We congratulate the authors for expanding knowledge on a challenging topic as the assessment of RV function in critically ill patients; indeed, the precise evaluation of RV performance in the intensive care unit is rather complex even in experienced hands [5, 6].

## Main text

However, several results of this study deserve comments and clarifications. All together with an exhaustive CCE assessment, the authors [1] reported the outcome according to the subtypes of ventricular dysfunction; in this regard, it appears that isolated RV dysfunction had the same mortality (33.3%) as compared to biventricular systolic dysfunction (31.6%). Furthermore, looking at the results of the multivariate analysis, the authors confirmed that LV systolic dysfunction had no significant impact on mortality (*p* = 0.52). The questionable impact of LV systolic dysfunction on outcome in sepsis has been previously confirmed by several meta-analyses [7–9]. Conversely, LV diastolic dysfunction was associated with 30-day mortality: Odds Ratio 2.38 (1.07–5.26; *p* = 0.03), which seems having a similar weight to the impact of RV dysfunction, Odds Ratio 2.01 (1.07–3.81, *p* = 0.03). Although this finding points in the same direction of previous studies suggesting the important role of LV diastolic dysfunction in the outcome of critically ill patients [9–11], also in the pediatric population [12, 13], it should be noted that an assessment relying on E/e’ ratio only would have been better discussed as likelihood of increased LV filling pressure [14, 15]. The use of E/e’ ratio together with e’ septal values has been suggested by Lanspa et al. [16, 17] to simplify the assessment of LV diastolic dysfunction in critically ill patients [18]. However, this assessment may not always provide interchangeable findings as compared to the guidelines used in the cardiology setting [19], as recently highlighted in patients with coronavirus disease-19 [20–22].

Other aspects of the work conducted by Bendary et al. [1] deserving comments are focused on strain echocardiography. Indeed, the authors reported in the methods they assessed both LV and RV strain. Subsequently, they report no differences in mortality according to values of LV global longitudinal strain while, interestingly, this parameter had been previously associated with greater mortality in septic patients [23]; moreover, it is commonly accepted that deterioration in LV performance is identified earlier by strain as compared to the use of more conventional parameters [24–26]. One possible explanation for this difference may be the very early assessment conducted by Bendary et al.[1] (within 24 h of sepsis presentation), suggesting that further research is needed on role of strain in critically ill patients [27]. In truth it must be also admitted that the meta-analysis suggesting association between LV global longitudinal strain and mortality in septic patients lacks of a trial sequential analysis [28], thus leaving uncertainty on the robustness of its findings. A second issue regarding strain is that authors assessed the RV strain measuring free wall but they did not actually report their results, leaving uncertainties on the role of RV strain in early identification of septic cardiomyopathy and on its role as predictor of outcome. Moreover, another uncertainty on the strain assessment conducted by Bendary et al. [1] regards the absence of data on its feasibility. It is well known that strain relies on very good image quality and this is not always achievable in critically ill patients, especially when ventilated with positive pressure. Therefore, reporting feasibility would be ideal when discussing strain echocardiography.

Finally, we may suggest the authors to adhere with the PRICES (*Preferred Reporting Items for Critical care Echocardiography research Studies*) recommendations [29], which were published with the aim to decrease reporting bias in CCE studies.

## Conclusions

Of note, the PRICES guidelines are not only based on experts’ opinion (panel of 20 experts) but also on an extensive systematic literature appraisal [30], and provide authors with checklists on the items that need reporting. Adherence to the PRICES guidelines is beneficial for the interpretation of the CCE study itself, reducing the risk of underreporting clinically meaningful information; additionally, adherence to PRICES may increase the study reproducibility and its comparison with future studies.

## Data Availability

Data sharing is not applicable to this article as no datasets were generated or analyzed during the current study.

## Abbreviations

CCE: Critical care echocardiography
RV: Right ventricular
LV: Left ventricular

## References

[1] Bendary A, Said H, Elemary M, Mahrous M (2022). Right ventricular function as a predictor of short-term mortality in patients with sepsis and septic shock: an observational study. Egypt Heart J (EHJ) Off Bull Egypt Soc Cardiol.

[2] Geri G, Vignon P, Aubry A, Fedou AL, Charron C, Silva S, Repesse X, Vieillard-Baron A (2019). Cardiovascular clusters in septic shock combining clinical and echocardiographic parameters: a post hoc analysis. Intensive Care Med.

[3] Sanfilippo F, Orde S, Oliveri F, Scolletta S, Astuto M (2019). The challenging diagnosis of septic cardiomyopathy. Chest.

[4] Vieillard-Baron A, Millington SJ, Sanfilippo F, Chew M, Diaz-Gomez J, McLean A, Pinsky MR, Pulido J, Mayo P, Fletcher N (2019). A decade of progress in critical care echocardiography: a narrative review. Intensive Care Med.

[5] Vieillard-Baron A, Naeije R, Haddad F, Bogaard HJ, Bull TM, Fletcher N, Lahm T, Magder S, Orde S, Schmidt G, Pinsky MR (2018). Diagnostic workup, etiologies and management of acute right ventricle failure : a state-of-the-art paper. Intensive Care Med.

[6] Orde S, Slama M, Yastrebov K, McLean A, Huang S (2019). Subjective right ventricle assessment by echo qualified intensive care specialists: assessing agreement with objective measures. Crit Care (London, England).

[7] Huang SJ, Nalos M, McLean AS (2013). Is early ventricular dysfunction or dilatation associated with lower mortality rate in adult severe sepsis and septic shock? A meta-analysis. Crit Care (London, England).

[8] Sanfilippo F, Huang S, Messina A, Franchi F, Oliveri F, Vieillard-Baron A, Cecconi M, Astuto M (2021). Systolic dysfunction as evaluated by tissue Doppler imaging echocardiography and mortality in septic patients: a systematic review and meta-analysis. J Crit Care.

[9] Sanfilippo F, Corredor C, Fletcher N, Landesberg G, Benedetto U, Foex P, Cecconi M (2015). Diastolic dysfunction and mortality in septic patients: a systematic review and meta-analysis. Intensive Care Med.

[10] Sanfilippo F, Di Falco D, Noto A, Santonocito C, Morelli A, Bignami E, Scolletta S, Vieillard-Baron A, Astuto M (2021). Association of weaning failure from mechanical ventilation with transthoracic echocardiography parameters: a systematic review and meta-analysis. Br J Anaesth.

[11] Sanfilippo F, Corredor C, Arcadipane A, Landesberg G, Vieillard-Baron A, Cecconi M, Fletcher N (2017). Tissue Doppler assessment of diastolic function and relationship with mortality in critically ill septic patients: a systematic review and meta-analysis. BJA Br J Anaesth.

[12] Sankar J, Das RR, Jain A, Dewangan S, Khilnani P, Yadav D, Dubey N (2014). Prevalence and outcome of diastolic dysfunction in children with fluid refractory septic shock–a prospective observational study. Pediatric Crit Care Med J Soc Crit Care Med World Fed Pediatric Intensive Crit Care Soc.

[13] Sanfilippo F, La Rosa V, Grasso C, Santonocito C, Minardi C, Oliveri F, Iacobelli R, Astuto M (2020). Echocardiographic parameters and mortality in pediatric sepsis: a systematic review and meta-analysis. Pediatric Crit Care Med J Soc Crit Care Med World Fed Pediatric Intensive Crit Care Soc.

[14] Kasner M, Westermann D, Steendijk P, Gaub R, Wilkenshoff U, Weitmann K, Hoffmann W, Poller W, Schultheiss HP, Pauschinger M, Tschope C (2007). Utility of Doppler echocardiography and tissue Doppler imaging in the estimation of diastolic function in heart failure with normal ejection fraction: a comparative Doppler-conductance catheterization study. Circulation.

[15] Sanfilippo F, Bignami EG, Astuto M, Messina A, Cammarota G, Maggiore SM, Vetrugno L (2022). Understanding left ventricular diastolic dysfunction in anesthesia and intensive care patients: "a glass with progressive shape change". Minerva Anestesiol.

[16] Brown SM, Pittman JE, Hirshberg EL, Jones JP, Lanspa MJ, Kuttler KG, Litwin SE, Grissom CK (2012). Diastolic dysfunction and mortality in early severe sepsis and septic shock: a prospective, observational echocardiography study. Crit Ultrasound J.

[17] Lanspa MJ, Gutsche AR, Wilson EL, Olsen TD, Hirshberg EL, Knox DB, Brown SM, Grissom CK (2016). Application of a simplified definition of diastolic function in severe sepsis and septic shock. Crit Care (London, England).

[18] Sanfilippo F, Scolletta S, Morelli A, Vieillard-Baron A (2018). Practical approach to diastolic dysfunction in light of the new guidelines and clinical applications in the operating room and in the intensive care. Ann Intensive Care.

[19] Nagueh SF, Smiseth OA, Appleton CP, Byrd BF, Dokainish H, Edvardsen T, Flachskampf FA, Gillebert TC, Klein AL, Lancellotti P, Marino P, Oh JK, Alexandru Popescu B, Waggoner AD (2016). Recommendations for the evaluation of left ventricular diastolic function by echocardiography: an update from the American Society of Echocardiography and the European Association of Cardiovascular Imaging. Eur Heart J Cardiovasc Imaging.

[20] La Via L, Dezio V, Santonocito C, Astuto M, Morelli A, Huang S, Vieillard-Baron A, Sanfilippo F (2022). Full and simplified assessment of left ventricular diastolic function in covid-19 patients admitted to ICU: Feasibility, incidence, and association with mortality. Echocardiography (Mount Kisco, NY).

[21] Messina A, Sanfilippo F, Milani A, Calabrò L, Negri K, Monge García MI, Astuto M, Vieillard-Baron A, Cecconi M (2021). COVID-19-related echocardiographic patterns of cardiovascular dysfunction in critically ill patients: a systematic review of the current literature. J Crit Care.

[22] Huang S, Vignon P, Mekontso-Dessap A, Tran S, Prat G, Chew M, Balik M, Sanfilippo F, Banauch G, Clau-Terre F, Morelli A, De Backer D, Cholley B, Slama M, Charron C, Goudelin M, Bagate F, Bailly P, Blixt PJ, Masi P, Evrard B, Orde S, Mayo P, McLean AS, Vieillard-Baron A (2022). Echocardiography findings in COVID-19 patients admitted to intensive care units: a multi-national observational study (the ECHO-COVID study). Intensive Care Med.

[23] Sanfilippo F, Corredor C, Fletcher N, Tritapepe L, Lorini FL, Arcadipane A, Vieillard-Baron A, Cecconi M (2018). Left ventricular systolic function evaluated by strain echocardiography and relationship with mortality in patients with severe sepsis or septic shock: a systematic review and meta-analysis. Crit Care (London, England).

[24] Vignon P, Huang SJ (2015). Global longitudinal strain in septic cardiomyopathy: the hidden part of the iceberg?. Intensive Care Med.

[25] Orde SR, Pulido JN, Masaki M, Gillespie S, Spoon JN, Kane GC, Oh JK (2014). Outcome prediction in sepsis: speckle tracking echocardiography based assessment of myocardial function. Crit Care (London, England).

[26] Sanfilippo F, Santonocito C, Panarello G, Arcadipane A (2016). The role of speckle tracking echocardiography for prognostication in patients with severe sepsis or septic shock. Criti Care (London, England).

[27] Mayo P, Arntfield R, Balik M, Kory P, Mathis G, Schmidt G, Slama M, Volpicelli G, Xirouchaki N, McLean A, Vieillard-Baron A (2017). The ICM research agenda on critical care ultrasonography. Intensive Care Med.

[28] La Via L, Messina S, Merola F, Sanfilippo F, Astuto M (2022). Trial sequential analysis of a recently published study on video laryngoscopy versus direct laryngoscopy for nasotracheal intubation in oro-maxillofacial surgery. Korean J Anesthesiol.

[29] Sanfilippo F, Huang S, Herpain A, Balik M, Chew MS, Clau-Terré F, Corredor C, De Backer D, Fletcher N, Geri G, Mekontso-Dessap A, McLean A, Morelli A, Orde S, Petrinic T, Slama M, van der Horst ICC, Vignon P, Mayo P, Vieillard-Baron A (2021). The PRICES statement: an ESICM expert consensus on methodology for conducting and reporting critical care echocardiography research studies. Intensive Care Med.

[30] Huang S, Sanfilippo F, Herpain A, Balik M, Chew M, Clau-Terré F, Corredor C, De Backer D, Fletcher N, Geri G, Mekontso-Dessap A, McLean A, Morelli A, Orde S, Petrinic T, Slama M, van der Horst ICC, Vignon P, Mayo P, Vieillard-Baron A (2020). Systematic review and literature appraisal on methodology of conducting and reporting critical-care echocardiography studies: a report from the European Society of Intensive Care Medicine PRICES expert panel. Ann Intensive Care.

